# Evolving Magnetic Resonance Imaging (MRI) Findings in Immune Effector Cell-Associated Neurotoxicity Syndrome

**DOI:** 10.7759/cureus.84407

**Published:** 2025-05-19

**Authors:** Mark D Marino, John P Mader, William Kardasis, Matthew Murphy, Shamseldeen Y Mahmoud

**Affiliations:** 1 Radiology, Saint Louis University School of Medicine, Saint Louis, USA

**Keywords:** car-t therapy, immune effector cell-associated neurotoxicity syndrome (icans), magnetic resonance imaging, multiple myeloma, neuroradiology

## Abstract

Immune effector cell-associated neurotoxicity syndrome (ICANS) is a potentially life-threatening complication often observed in patients receiving immunotherapies like chimeric antigen receptor T-cell (CAR-T) therapy. This case report is based on a chart review of the history, physical examination of the primary team, laboratory tests, imaging findings, and discharge summary of the patient's hospital admission and subsequent encounters. We present the case of a middle-aged man with multiple myeloma who developed ICANS following two cycles of talquetamab (Talvey) therapy. The patient's initial symptoms included fever and altered mental status. Magnetic resonance imaging (MRI) revealed a multitude of foci, including T1 enhancement of the hypothalamus and caudate heads, fluid-attenuated inversion recovery (FLAIR) hyperintensities of the caudate nuclei and subependymal regions, susceptibility artifacts in the lentiform nuclei, and diffusion restriction in the corpus callosum. Altered mental status, fever, and elevations in cerebrospinal protein raised concern for possible neurotoxicity, and the patient was treated with steroids and tocilizumab. A follow-up MRI taken 24 days later demonstrated a progression of signal abnormalities in the caudate nuclei and a new rim of susceptibility in the bilateral caudate heads. A third MRI was performed 99 days after the original MRI, which tracked the resolution of the diffusion restriction and FLAIR abnormalities, but noted further increased susceptibility artifacts and new peripherally enhancing lesions in the bilateral caudate heads. A final MRI taken 250 days after the initial MRI tracked the resolution of the caudate lesions, with stable susceptibility artifacts. This case highlights the evolving nature of ICANS and underscores the role of serial MRI in tracking neurotoxicity progression, guiding treatment, and improving outcomes in immunotherapy-related complications.

## Introduction

Immune effector cell-associated neurotoxicity syndrome (ICANS) is a potentially life-threatening neurologic reaction observed in patients receiving immunotherapies, particularly chimeric antigen receptor T-cell (CAR-T) therapy for refractory lymphoma and multiple myeloma. ICANS can present with symptoms of mild confusion, lethargy, headache, difficulty concentrating, aphasia, focal neurologic deficits, tremor, encephalopathy, and seizures [1]. The American Society of Transplantation and Cellular Therapy states that ICANS is a clinical diagnosis and neurotoxicity is graded on a scale from 1 to 4 using the Common Terminology Criteria for Adverse Events [1]. Studies estimate that 20-70% of patients treated with CAR-T develop ICANS [2]. The exact mechanism of ICANS remains under investigation, but it is largely attributed to the inflammatory cascade involving pro-inflammatory cytokines such as IL-1, IL-6, and IFN-γ, which disrupt the blood-brain barrier. Researchers have proposed a multitude of possible cerebrospinal fluid (CSF) laboratory markers associated with ICANS; increased CSF protein and white blood cells are commonly seen in clinical practice and can contribute to the clinical diagnosis [3]. Magnetic resonance imaging (MRI) is the ideal modality to detect brain abnormalities from ICANS. MRI results may show signs of cerebral edema, particularly in advanced disease, which correlates with more severe symptoms such as intractable seizures and coma. A common finding is white matter changes presenting as T2/fluid-attenuated inversion recovery (FLAIR) hyperintensities, particularly in the corpus callosum or basal ganglia, as reported in some cases [2-5]. Diffusion abnormalities and microhemorrhages have been noted in several locations in other case reports. However, there is a sparsity of research on the evolution of these findings over time. This case presentation outlines the development of several brain abnormalities in a patient diagnosed with ICANS who underwent serial brain MRI to track the progression and resolution of these neuroimaging findings. 

## Case presentation

**Figure 1:**
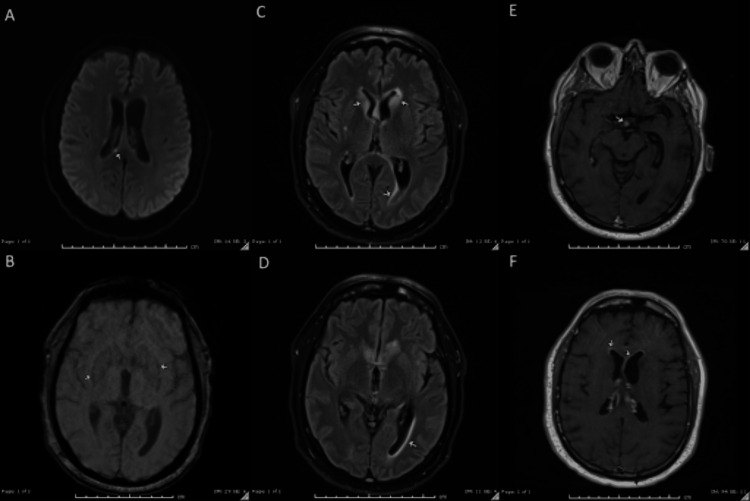
Initial MRI findings (A) DWI: An area of diffusion restriction within the superior right splenium measuring 3 mm. (B) SWI: A subtle rim of susceptibility surrounding the bilateral lentiform nuclei. (C-D) FLAIR: Periventricular hyperintensities are seen surrounding the frontal horns of the lateral ventricles, with involvement of the bilateral caudate nuclei, left more than right. There are an additional mild hyperintensity surrounding the left trigone and occipital horn and a minimal hyperintensity surrounding the right trigone and occipital horn. No significant mass effect secondary to this edema. Scattered hyperintensities throughout the cortical white matter likely represent small vessel ischemic changes. (E-F) T1 with contrast: A 6-mm area of enhancement within the region of the hypothalamus. There is curvilinear enhancement along the ependymal surface of the frontal horns of the lateral ventricles. MRI: magnetic resonance imaging; DWI: diffusion-weighted imaging; SWI: susceptibility-weighted imaging; FLAIR: fluid-attenuated inversion recovery

Recently, a middle-aged man with a history of multiple myeloma treated with two cycles of talquetamab (Talvey), metabolic dysfunction-associated steatotic liver disease, and hypertension presented with altered mental status and fevers. He was seen in the clinic to start his third cycle of Talvey, but his wife reported he was experiencing fevers up to 102°F despite acetaminophen use and confusion at home for the past 24 hours. In the clinic, he was afebrile and denied any headache, double vision, nausea, or vomiting. On physical exam, he was alert and oriented to person, place, and time, without any focal neurological deficits, but demonstrated impaired short-term memory. He was admitted to the bone marrow transplant service for neurostatus observation, and Talvey was permanently discontinued due to concerns for potential neurotoxicity. Infectious disease was consulted for fever in an immunocompromised patient. Neurosurgery was consulted due to concern for brain metastasis. The initial MRI brain with and without contrast demonstrated several concerning foci in the caudate nuclei, corpus callosum, hypothalamus, and subependymal regions (Figure 1). Enhancing lesions within the calvaria were unchanged from prior imaging and attributed to previously diagnosed multiple myeloma. 

Blood culture results were negative. All serological and cerebrospinal infectious workup was negative. However, CSF studies did reveal xanthochromia, an elevated white cell count, and an elevated protein level of 285 mg/dL (Table 1).

**Table 1:** A summary of notable lab findings with reference ranges included Abnormal results are bolded. CSF: cerebrospinal fluid; RBC: red blood cell; CF: complement fixation; CMV: cytomegalovirus; PCR: polymerase chain reaction

Lab	Reference range	Patient values
White blood cells	4-10.7 g/dL	4.4
Total cell CSF	0-5 cells/µL	100
Nucleated cell CSF	0-5 cells/µL	125
RBC CSF	0 cells/µL	12
Xanthochromia CSF	Absent	Present
Glucose CSF	40-70 mg/dL	51
IgG CSF	0-6 mg/dL	35.6
*Cryptococcus* antigen CSF	Negative	Negative
Protein CSF	5–45 mg/dL	285
*Aspergillus* antigen BAL/serum	0-0.49	0.09
*Coccidioides* antibody CF	<1:2	<1:2
*Histoplasma *antibody	Not detected	Not detected
*Histoplasma *yeast CF antibody	<1:8	<1:8
CMV quantitative PCR	Not detected	Not detected
Herpes simplex virus 2 PCR CSF	Not detected	Not detected
Human herpesvirus 6	Not detected	Not detected
Human parechovirus	Not detected	Not detected
Varicella zoster virus	Not detected	Not detected

Given concern for cytokine release syndrome or ICANS, the patient was started on dexamethasone every six hours and received one dose of tocilizumab. He remained neurologically stable, and steroids were tapered. He was discharged home awaiting cerebrospinal cytology, which later resulted negative for malignant cells. Two weeks after discharge from the hospital, the patient had a witnessed generalized tonic-clonic seizure and recovered after two minutes. He did not seek emergent care at that time, but notified his oncologist at his next clinic appointment, and was started on levetiracetam. No further seizure activity was documented. 

**Figure 2:**
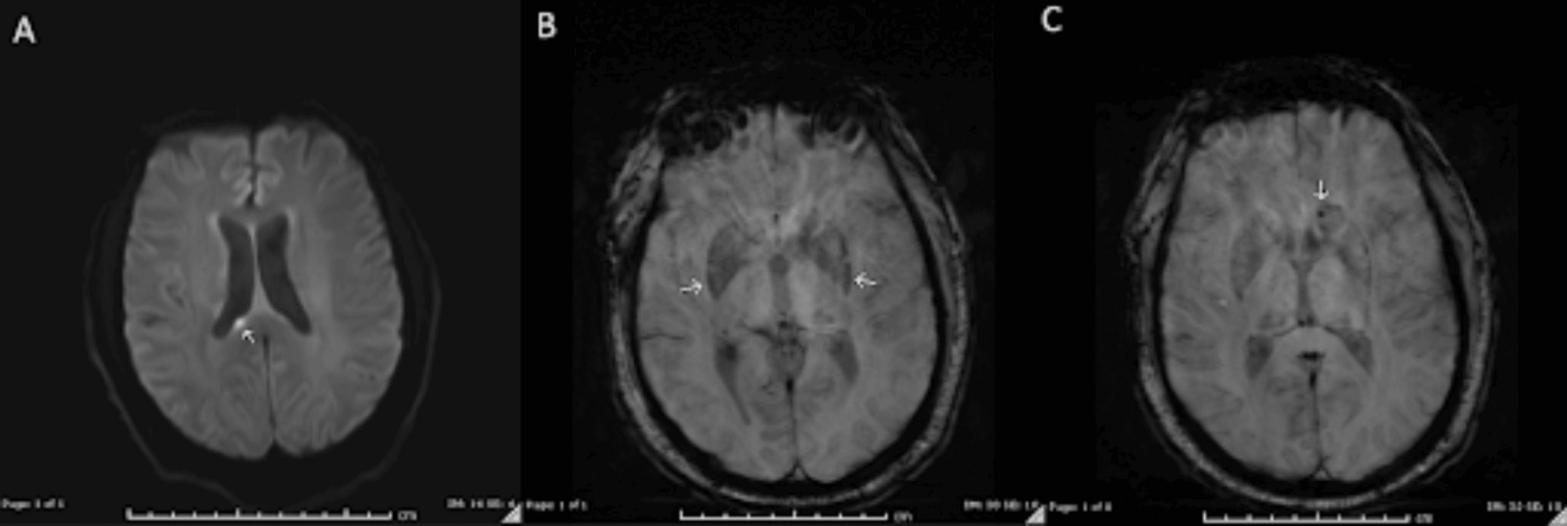
Repeat MRI 24 days after the original MRI (A) DWI: Area of diffusion restriction within the superior right splenium now measures 4 mm, unchanged accounting for differences in technique and measurement. (B-C) SWI: Increased conspicuity of a rim of susceptibility surrounding the left lentiform nucleus with interval development of more discrete foci of susceptibility within the head of the caudate nucleus on the left likely representing the interval development of microhemorrhages within the head of the caudate. MRI: magnetic resonance imaging; DWI: diffusion-weighted imaging; SWI: susceptibility-weighted imaging

**Figure 3:**
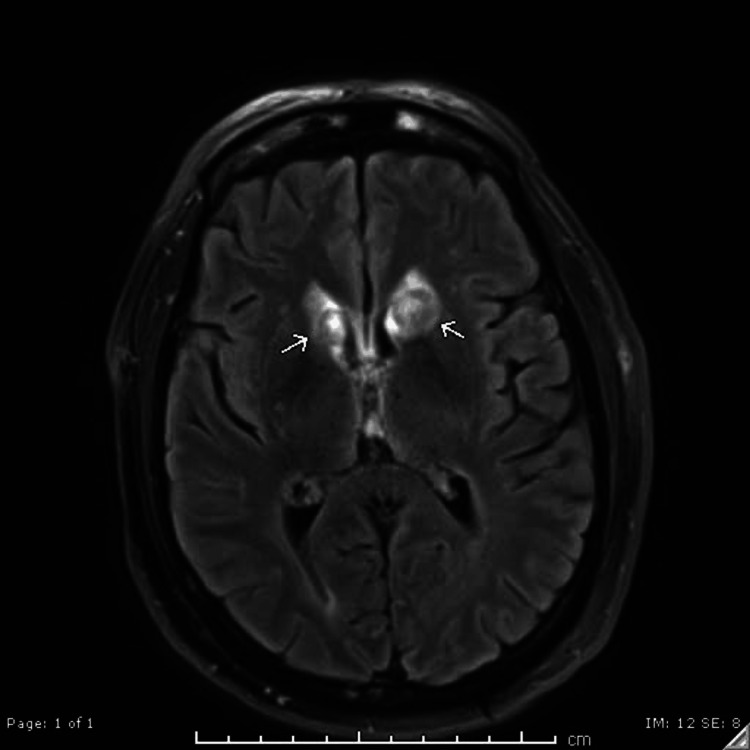
MRI FLAIR sequence 24 days after the original MRI Increased signal abnormalities within the bilateral caudate nuclei. The FLAIR signal within the bilateral caudate lobes appears more heterogeneous with mixed hypointensity and hyperintensity with the rim of hypointensity surrounding a central focus of hyperintensity with an outermost rim of additional hyperintensity. Similar to prior, the left caudate nucleus is more affected than the right with the left measuring 1.5 times the size of the right. Interval development of mass effect from this increased edema resulting in mild mass effect/compression of the frontal horns. Decreased hyperintensities surrounding the bilateral trigones and occipital horns when compared to prior. MRI: magnetic resonance imaging; FLAIR: fluid-attenuated inversion recovery

**Figure 4:**
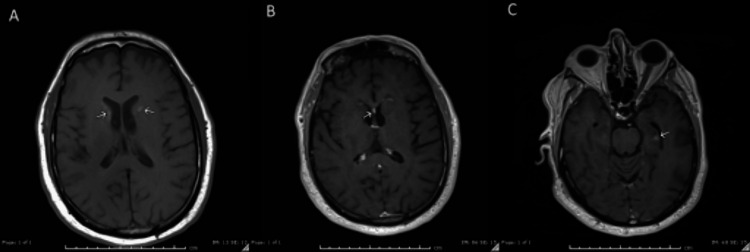
MRI T1 with and without contrast enhancement 24 days after the original MRI (A) T1 without contrast enhancement showing interval increased size of signal abnormalities involving the bilateral caudate nuclei. (B) T1 with contrast enhancement showing interval development of a 5-mm area of enhancement of the septum pellucidum. (C) T1 with contrast enhancement showing interval development of a small focus of patchy ill-defined enhancement within the region of the left hippocampus measuring 6 mm. MRI: magnetic resonance imaging

Twenty-four days after the initial MRI, the patient underwent repeat imaging. The previously noted diffusion restriction within the superior right splenium remained stable in size (4 mm), and there was interval resolution of a 6-mm focus of susceptibility artifact of the stalk of the pituitary (Figure 2A-2C). Susceptibility-weighted imaging revealed an increased conspicuity of a susceptibility rim surrounding the left lentiform nucleus, along with newly discrete microhemorrhages within the left caudate head (Figure 2B-2C). FLAIR sequences demonstrated progression of signal abnormalities within the bilateral caudate nuclei, with a heterogeneous pattern of hypointensity and hyperintensity (Figure 3). The left caudate remained more affected, 1.5 times larger than the right, contributing to mild mass effect on the frontal horns. There was also a decrease in FLAIR hyperintensities surrounding the bilateral trigones and occipital horns compared to prior imaging. T1 sequences showed resolution of the curvilinear ependymal focus of enhancement along the frontal lateral horns. However, there was interval development of foci at the bilateral heads of the caudate (Figure 4A), the septum pellucidum (Figure 4B), and the left hippocampus (Figure 4C).

**Figure 5:**
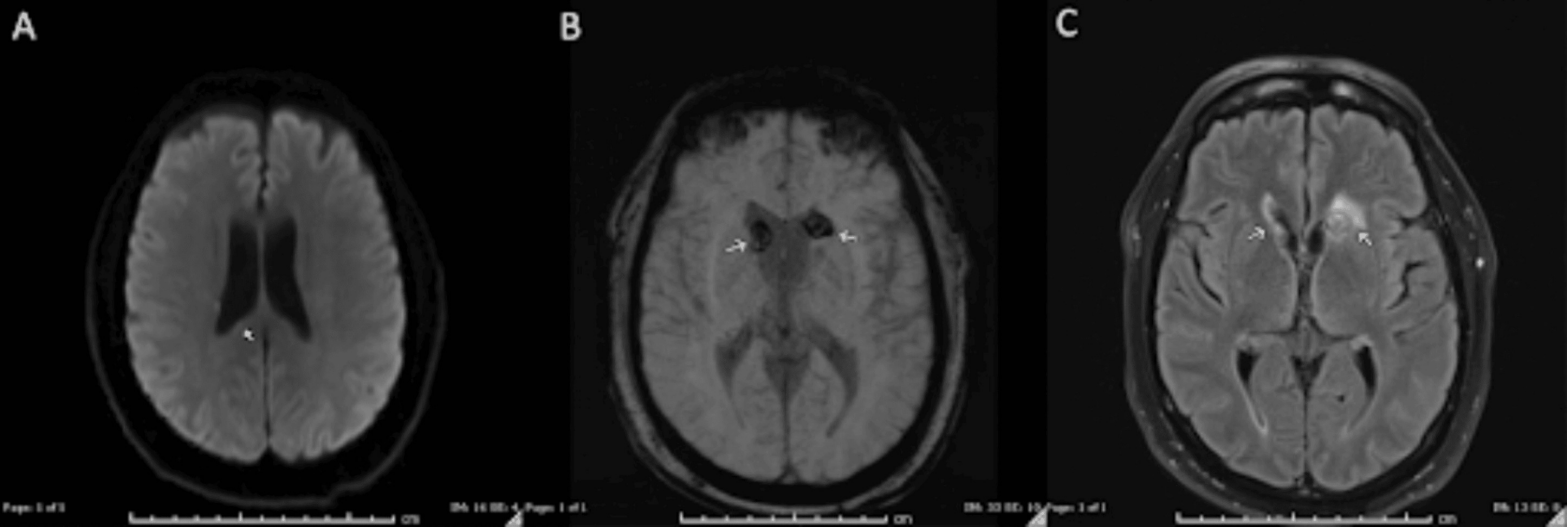
Repeat MRI 99 days after the original MRI (A) DWI: Interval near-complete resolution of the focus of restricted diffusion in the right aspect of the splenium of the corpus callosum. (B) SWI: Interval enlargement of susceptibility artifacts in the caudate heads compatible with hemosiderin deposition/underlying hemorrhage. (C) FLAIR: Decreased abnormal FLAIR signal in the caudate heads. MRI: magnetic resonance imaging; DWI: diffusion-weighted imaging; SWI: susceptibility-weighted imaging; FLAIR: fluid-attenuated inversion recovery

**Figure 6:**
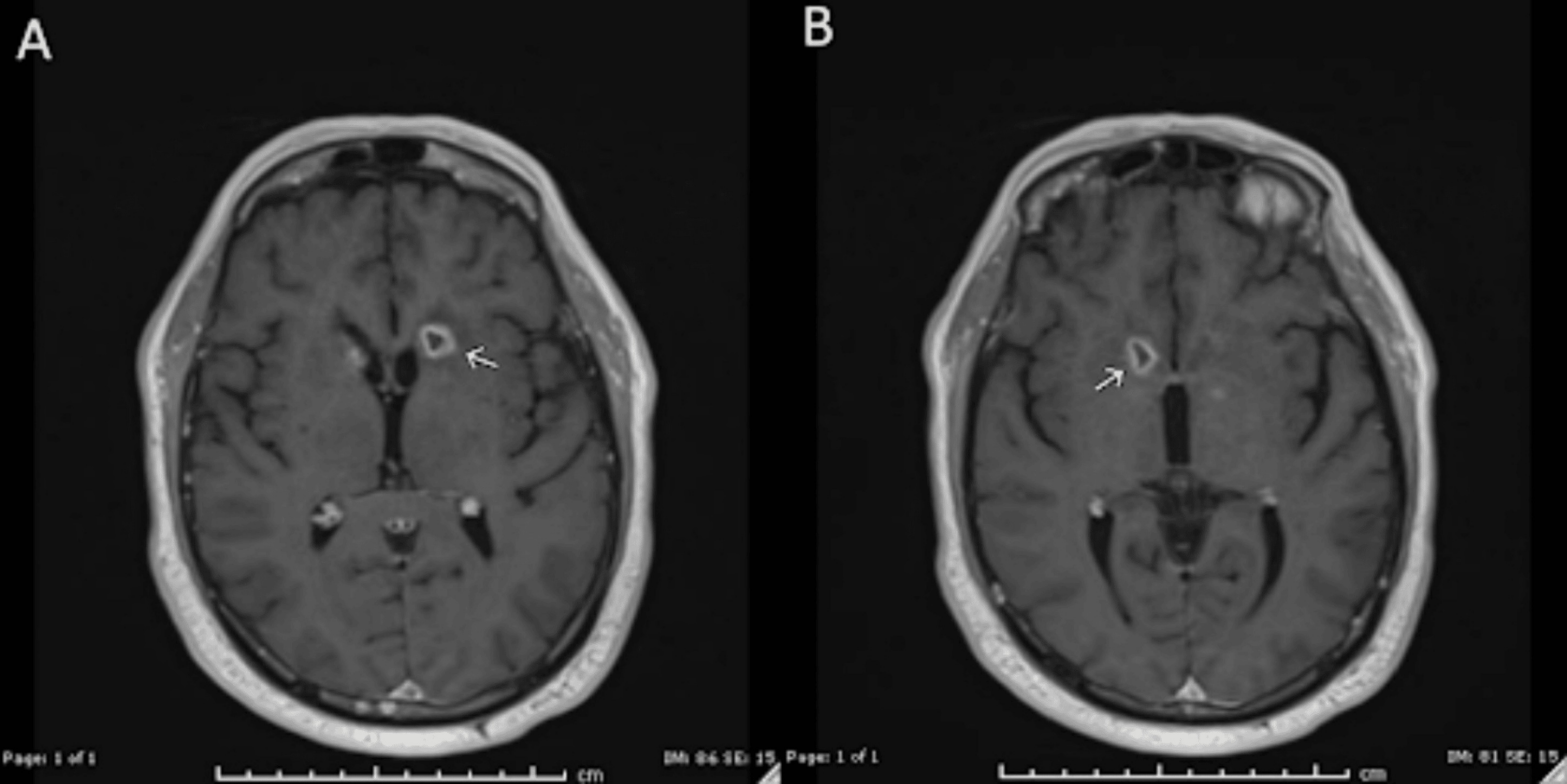
MRI T1 with contrast enhancement 99 days after the original MRI (A-B) Interval development of peripherally enhancing lesions in the bilateral caudate heads. MRI: magnetic resonance imaging

Ninety-nine days after the initial MRI, the patient underwent another repeat MRI to monitor the previously described abnormalities. Diffusion restriction in the right splenium had nearly resolved (Figure 5A). Susceptibility artifacts previously seen in the lentiform nuclei had resolved, and new susceptibility artifacts emerged in the caudate heads (Figure 5B). While FLAIR lesions in the caudate heads were decreased in size (Figure 5C), enlarged peripherally enhancing lesions in the bilateral caudate heads were discovered (Figure 6).

**Figure 7:**
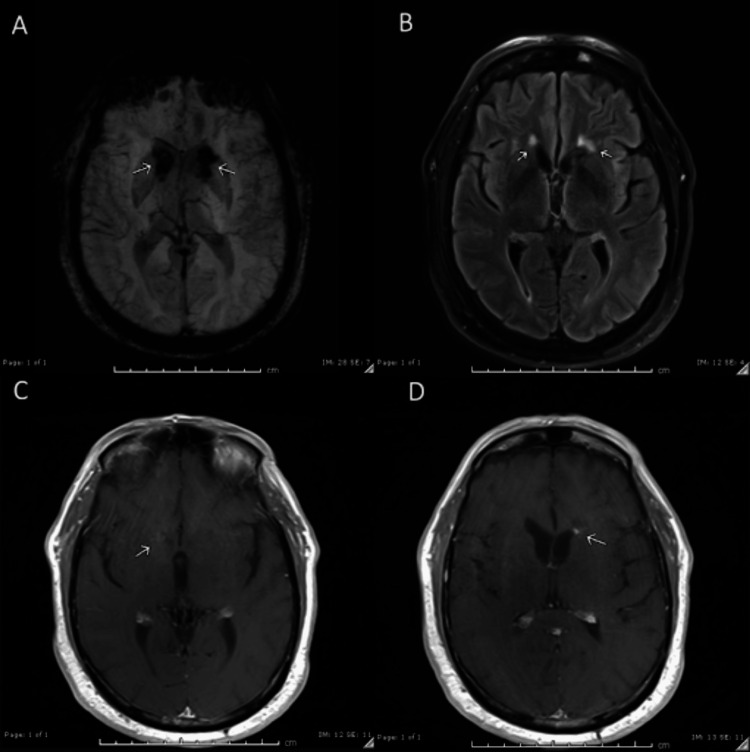
Repeat MRI 250 days after the original MRI (A) SWI: Persistent, possibly increased susceptibility artifacts in the caudate heads compatible with hemosiderin deposition/underlying hemorrhage, unclear whether related to differences in the imaging techniques. (B) FLAIR: Continued interval decreased size of the signal abnormalities involving the caudate nuclei. (C-D) T1 with contrast enhancement: Interval resolution of the enhancement in the right caudate head and significantly decreased enhancement in the left caudate head. MRI: magnetic resonance imaging; SWI: susceptibility-weighted imaging; FLAIR: fluid-attenuated inversion recovery

Lastly, 250 days after the initial MRI, the patient underwent another MRI to track the progression of these imaging findings (Figure 7). This final MRI demonstrated stable, possibly increased, susceptibility artifacts in the bilateral caudate heads and interval decreased size of the caudate head lesions previously seen on FLAIR and T1 sequences. At this time, the patient has not had further seizure activity or episodes of altered mental status, but continues to report occasional episodes of forgetfulness, which were not present before he was diagnosed with ICANS.

## Discussion

This case highlights the evolving clinical and neuroimaging findings of ICANS. Symptoms of fever and confusion in an immunocompromised patient necessitated an infectious workup, but also created concern for cytokine release syndrome and low-grade ICANS. CSF studies resulted in several nonspecific markers for ICANS. When combined with the clinical picture in this case, neuroimaging can confidently correlate with the development of neurotoxicity secondary to CAR-T therapy. 

This patient's initial presentation with altered mental status and fever would typically correlate to grade 1 ICANS. While this patient later suffered a generalized tonic-clonic seizure several weeks after receiving treatment for ICANS, a single brief generalized seizure episode still leads to a low-grade (grade 2) ICANS diagnosis [1]. Reports in the literature, albeit with small sample sizes, state that a majority of patients with low-grade ICANS have normal MRI findings [6]. This case is particularly interesting given the multitude of severe imaging findings, including cerebral edema and mass effect, on MRI that indicate neurotoxicity despite this patient only experiencing low-grade symptoms of ICANS. The delayed onset of seizure provides a clinical sign that the neurotoxicity of ICANS can progress long after CAR-T therapy is discontinued, which was also proven by serial imaging. This case demonstrates the clinical utility of MRI in diagnosing neurotoxicity when symptoms and laboratory results are unrevealing or nonspecific. 

The MRI findings in this case illustrate a dynamic progression of neurotoxicity in ICANS following talquetamab immunotherapy. A small stable focus of restricted diffusion in the splenium of the corpus callosum, though nonspecific, aligns with known patterns of neuroinflammation in ICANS and requires continued monitoring. The development of susceptibility artifacts in the lentiform and caudate nuclei correlates with microhemorrhage or hemosiderin deposition over time in the setting of neuroinflammation. Increased susceptibility artifacts up to 250 days after the initial MRI provide rare evidence of chronic injury due to ICANS. These findings may correlate to continued damage to the blood-brain barrier in ICANS. The increased size and heterogeneity of signal abnormalities in the caudate nuclei, even 99 days later, suggest sequelae of chronic neuroinflammation. The development and evolution of caudate hyperintensity is not a hallmark of ICANS. The involvement of the caudate nuclei may correlate with neurological symptoms like confusion, memory loss, and motor dysfunction. Importantly, the resolution of the foci in the hypothalamus, ependymal regions, bilateral trigones, and occipital horns points to partial recovery, while new hyperattenuation in the septum pellucidum, hippocampus, and caudate nuclei indicates ongoing inflammation and demonstrates the continued impact of ICANS. The delayed development of new lesions in the hippocampus is concerning given their potential impact on cognition and memory. These findings underscore the importance of long-term serial imaging in assessing the extent of neurotoxicity and guiding treatment in ICANS. Additional research should be devoted to developing consensus for physicians interpreting neuroimaging of ICANS patients to improve diagnostic accuracy, enhance the scientific understanding of the pathophysiology of ICANS, and improve the generalizability of the findings reported in this single-patient case report.

Given the nonspecific nature of many neuroimaging findings associated with ICANS, some medical societies recommend obtaining a baseline MRI for patients starting talquetamab to differentiate acute from chronic findings [7]. This case opens further discussion into the clinical utility in comparing the brain during acute inflammation to a pretreatment baseline or if serial neuroimaging alone is sufficient to track treatment response and regression of inflammatory changes in ICANS. The evolution of brain abnormalities over the course of months in this case of ICANS highlights the importance of long-term serial imaging, even if some initial signal abnormalities resolve in the subacute phase. It is difficult to make broad generalizations about the diagnosis and management of ICANS based on this single case report. However, these novel imaging findings warrant additional study into the subacute and chronic progression of neurotoxicity and the role of MRI in monitoring ICANS. 

## Conclusions

This case highlights the dynamic and evolving nature of MRI findings in ICANS in the months following CAR-T immunotherapy. The progression of caudate lesions, emergence of new hippocampal hyperattenuation, and development of cerebral edema with mass effect underscore the potential severity of neuroinflammation in ICANS, even in low-grade cases. This case is unique in that serial imaging demonstrated subacute and chronic neurotoxicity despite adequate treatment. Furthermore, this case reveals delayed development of inflammation and microhemorrhage in the caudate nuclei, which is not commonly associated with ICANS. Meanwhile, the resolution of hypothalamic and ependymal foci suggests that some inflammatory changes are reversible with appropriate and prompt medical management. Serial MRI was critical in tracking these changes. As ICANS continues to be a serious and common complication of immunotherapies, recognizing key MRI patterns can aid in early diagnosis, improving patient outcomes in the evolving field of CAR-T therapy.

## References

[1] Lee DW, Santomasso BD, Locke FL (2019). ASTCT consensus grading for cytokine release syndrome and neurologic toxicity associated with immune effector cells. Biol Blood Marrow Transplant.

[2] Sterner RC, Sterner RM (2022). Immune effector cell associated neurotoxicity syndrome in chimeric antigen receptor-T cell therapy. Front Immunol.

[3] Gust J, Ponce R, Liles WC, Garden GA, Turtle CJ (2020). Cytokines in CAR T cell-associated neurotoxicity. Front Immunol.

[4] Lapidus AH, Anderson MA, Harrison SJ, Dickinson M, Kalincik T, Lasocki A (2022). Neuroimaging findings in immune effector cell associated neurotoxicity syndrome after chimeric antigen receptor T-cell therapy. Leuk Lymphoma.

[5] Holtzman NG, Xie H, Bentzen S (2021). Immune effector cell-associated neurotoxicity syndrome after chimeric antigen receptor T-cell therapy for lymphoma: predictive biomarkers and clinical outcomes. Neuro Oncol.

[6] Valand HA, Huda F, Tu RK (2019). Chimeric antigen receptor T-cell therapy: what the neuroradiologist needs to know. AJNR Am J Neuroradiol.

[7] de Groot PM, Arevalo O, Shah K (2022). Imaging primer on chimeric antigen receptor T-cell therapy for radiologists. Radiographics.

